# Public Health Lessons Learned From Biases in Coronavirus Mortality Overestimation

**DOI:** 10.1017/dmp.2020.298

**Published:** 2020-08-12

**Authors:** Ronald B. Brown

**Affiliations:** School of Public Health and Health Systems, University of Waterloo, Waterloo, Canada

**Keywords:** case fatality rate, coronavirus mortality overestimation, COVID-19, infection fatality rate, sampling bias

## Abstract

In testimony before US Congress on March 11, 2020, members of the House Oversight and Reform Committee were informed that estimated mortality for the novel coronavirus was 10-times higher than for seasonal influenza. Additional evidence, however, suggests the validity of this estimation could benefit from vetting for biases and miscalculations. The main objective of this article is to critically appraise the coronavirus mortality estimation presented to Congress. Informational texts from the World Health Organization and the Centers for Disease Control and Prevention are compared with coronavirus mortality calculations in Congressional testimony. Results of this critical appraisal reveal information bias and selection bias in coronavirus mortality overestimation, most likely caused by misclassifying an influenza infection fatality rate as a case fatality rate. Public health lessons learned for future infectious disease pandemics include: safeguarding against research biases that may underestimate or overestimate an associated risk of disease and mortality; reassessing the ethics of fear-based public health campaigns; and providing full public disclosure of adverse effects from severe mitigation measures to contain viral transmission.

On September 23, 1998, the US National Aeronautics and Space Administration (NASA) permanently lost contact with the $125 million Mars Climate Orbiter.^1^ A simple miscalculation, failure to convert English measurements to metric measurements, doomed the Mars space mission.^2^ A later investigation found that backup quality assurance procedures were not in place at NASA to catch and correct this simple miscalculation. Fast forward 22 years to another crisis involving a US government agency: On March 11, 2020, the US Congress House Oversight and Reform Committee received information from the National Institute of Allergy and Infectious Diseases (NIAID) concerning the novel coronavirus, severe acute respiratory syndrome coronavirus 2 (SARS-CoV-2), and coronavirus-disease 2019 (COVID-19).^3^ Based on the data available at the time, Congress was informed that the estimated mortality rate for the coronavirus was 10-times higher than for seasonal influenza, which helped launch a campaign of social distancing, organizational and business lockdowns, and shelter-in-place orders.

Previous to the Congressional hearing, a less severe estimation of coronavirus mortality appeared in a February 28, 2020 editorial released by NIAID and the Centers for Disease Control and Prevention (CDC). Published online in the New England Journal of Medicine (NEJM.org), the editorial stated:

“…the overall clinical consequences of Covid-19 may ultimately be more akin to those of a severe seasonal influenza (which has a case fatality rate of approximately 0.1%).”^4^

Almost as a parenthetical afterthought, the NEJM editorial inaccurately stated that 0.1% is the approximate case fatality rate of seasonal influenza. By contrast, the World Health Organization (WHO) reported that 0.1% or lower is the approximate influenza infection fatality rate,^5^ not the case fatality rate. To fully appreciate the significance of discrepancies in fatality rate usage by NIAID, the CDC, and the WHO, brief definitions of relevant epidemiological terms follow.

Case fatality rates (CFRs), infection fatality rates (IFRs), and mortality rates are used by epidemiologists to describe deaths during and after an infectious disease outbreak. The CDC defined a mortality rate as the frequency of deaths within a time period relative to the size of a well-defined population.^6^ Patients may be classified as having an influenza-like illness (ILI) such as COVID-19 according to standard criteria in a case definition.^7^ A CFR is defined as the proportion of deaths among confirmed cases of the disease. CFRs indicate the disease severity, while an IFR is defined as the proportion of deaths relative to the prevalence of infections within a population.^8^ IFRs are estimated following an outbreak, often based on representative samples of blood tests of the immune system in individuals exposed to a virus. Estimation of the IFR in COVID-19 is urgently needed to assess the scale of the coronavirus pandemic.^9^

Because different types of fatality rates can vary widely, it is imperative to not confuse fatality rates with one another; else misleading calculations with significant consequences could result. As of late spring 2020, a search of the keyword term “infection fatality rate” on the CDC website returned no matching results or similar terms, nor was the epidemiological term located in the 511-page CDC publication, *Principles of Epidemiology in Public Health Practice*. (The CDC eventually introduced the Infection Fatality Ratio (IFR) on July 10, 2020 “as a new parameter value for disease severity.”^10^) This terminology omission, in conjunction with questionable use of fatality rate terminology in the NEJM editorial, raises red flags, warning of possible inaccuracies in the coronavirus mortality estimation presented to Congress. Similar to the need to vet for miscalculations that might have rescued NASA’s 1998 Mars mission, vetting the coronavirus mortality estimation for miscalculations and biases may benefit the validity of mortality conclusions. Therefore, the purpose of this article is to present an ad hoc critical appraisal of the coronavirus mortality estimation presented to US Congress on March 11, 2020.

## MAIN

Findings from a comparative analysis of selected video and texts are used in this article to critically appraise the validity of coronavirus mortality calculations presented in US Congressional testimony. Critical appraisal is a process that judges the validity of scientific research evidence.^11^ Comparative analysis is a tool used in a grounded theory methodology to investigate an unexplored area through logical induction of coherent themes and explanations that are grounded in empirical evidence.^12^ Text from the February 2020 NEJM.org editorial and video of Congressional testimony are compared with reliable informational texts from the WHO and CDC. Inconsistencies, inaccuracies, biases, utilization, and consequences of the coronavirus mortality estimation are discussed.

In NIAID testimony before the House Oversight and Reform Committee Hearing on Coronavirus response, Day 1,^3^ the Committee learned that mortality from seasonal influenza is 0.1%. Additionally, it was reported to Congress that the overall coronavirus mortality of approximately 2-3% had been reduced to 1% to take into account infected people who are asymptomatic or have mild symptoms. The adjusted mortality rate from coronavirus of 1% was then compared with the 0.1% mortality rate from seasonal influenza, and the conclusion was reported to the House Committee that the coronavirus was 10-times more lethal than seasonal influenza.

In a comparative analysis with WHO and CDC documents, the coronavirus mortality rate of 2-3% that was adjusted to 1% in Congressional testimony is consistent with the coronavirus CFR of 1.8-3.4% (median, 2.6%) reported by the CDC.^13^ Furthermore, the WHO reported that the CFR of the H1N1 influenza virus (1918) is also 2-3%,^14^ similar to the unadjusted 2-3% CFR of the coronavirus reported in Congressional testimony, with no meaningful difference in mortality. As previously mentioned, the WHO also reported that 0.1% is the IFR of seasonal influenza,^5^ not the CFR of seasonal influenza as reported in the NEJM editorial.

## DISCUSSION

Confusion between CFRs and IFRs may seem trivial, and it is easy to overlook at first, but this confusion may have ultimately led to an unintentional miscalculation in coronavirus mortality estimation. IFRs from samples across the population include undiagnosed, asymptomatic, and mild infections, and are often lower compared with CFRs, which are based exclusively on relatively smaller groups of moderately to severely ill diagnosed cases at the beginning of an outbreak. Due to host defense mechanisms and autoimmunity provided by innate and adaptive immune responses,^15^ asymptomatic infections are often prevalent in influenza.^16^ With many asymptomatic infections already identified in COVID-19,^17^ it appears unlikely that the IFR in an ILI like COVID-19 would approximate the disease’s CFR. Presymptomatic infections can also lower the proportion of asymptomatic infections. For example, a CDC report found that asymptomatic individuals identified through reverse transcriptase-polymerase chain reaction (RT-PCR) testing developed symptoms a week later, and those individuals were re-classified as having been presymptomatic at the time of testing.^18^

**FIGURE 1:**
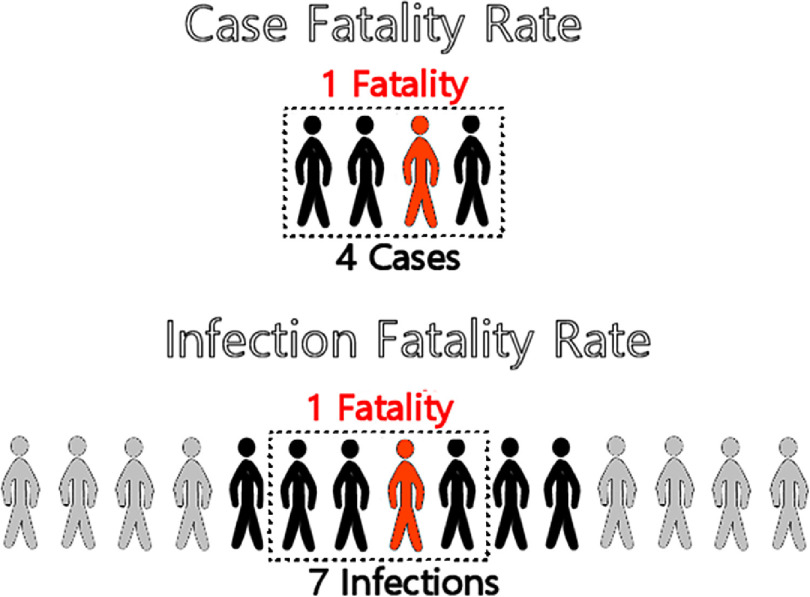
CFR and IFR. 1 fatality / 4 cases = 25% CFR. 1 fatality / 7 infections = 14.28% IFR.

In Figure 1, 4 cases grouped in the dotted-line box are also included among 7 infections, illustrating that all cases are infections but not all infections are cases, a potential point of confusion in media reports of COVID-19. For example, a high number of coronavirus infections were discovered in US meat-packing plants in Iowa,^19^ but these infections were reported as cases in the media,^20^ potentially causing a type of information bias known as misclassification.^21^ Misclassification refers to “the erroneous classification of an individual, a value, or an attribute into a category other than that to which it should be assigned.”^22^ This type of information bias in epidemiological research can lead to underestimates or overestimates of associated disease and mortality risks.^21^

CFRs and IFRs represent different segments of a targeted population and contain widely different proportions of nonfatal infections; therefore, misapplying findings or generalizing inferences between these 2 groups can cause a type of selection bias known as sampling bias^23^ or ascertainment bias.^24^ In this type of bias, people do not represent segments of the population to whom findings apply. Furthermore, “…comparisons of the CFR of 1 disease with the IFR of another are mostly useless,”^25^ and sampling bias can lead to serious inaccuracies, as when Congress was informed that the coronavirus is 10-times more lethal than seasonal influenza.

A comparison of coronavirus and seasonal influenza CFRs may have been intended during Congressional testimony, but due to misclassifying an IFR as a CFR, the comparison turned out to be between an adjusted coronavirus CFR of 1% and an influenza IFR of 0.1%. Had the adjusted coronavirus mortality rate not been lowered from 3% to 1%, fatality comparisons of the coronavirus to the IFR of seasonal influenza would have increased from 10-times higher to 20- to 30-times higher. By then, epidemiologists might have been alerted to the possibility of a miscalculation in such an alarming estimation.

### Quality Assurance

Most people rely on trusted public health experts from organizations like the CDC to disseminate vital information on infectious diseases.^26^ Unfortunately, even experts can make simple miscalculations that can lead to catastrophic results. In the example of NASA’s lost Mars Climate Orbiter, the NASA board investigating the failed mission recognized that mistakes happen on projects, “However, sufficient processes are usually in place on projects to catch these mistakes before they become critical to mission success.”^2^ The NASA board also recognized the importance of quality assurance procedures to prevent future failures. Of relevance, in 2018, the National Institute of Neurological Disorders and Stroke (NINDS) provided an exemplary definition of quality assurance (QA) in clinical and health sciences:

“The objectives of QA procedures are to assure the accuracy and consistency of study data, from the original observations through the reporting of results and to ensure that study results are considered valid and credible within the scientific and clinical communities.”^27^

Similar to NASA’s quality assurance problems in 1998, quality assurance procedures at US national public health organizations in 2020 may benefit from review and revision to prevent crucial mortality miscalculations of infectious diseases in the future. As a safeguard against misuse of fatality rates, and protection in the event of nonstandardized or inter-organizational discrepancies in terminology, every fatality rate should clearly define the denominator of the rate as the specific group to whom fatalities apply, either to the total population in mortality rates, confirmed cases of a disease in CFRs, or individuals exposed to a viral infection in IFRs.

### Mitigation Measures

As the campaign to mitigate coronavirus transmission was implemented from March into May, 2020, expected coronavirus mortality totals in the United States appeared much lower than the overestimation reported in Congressional testimony on March 11. Compared with the most recent season of severe influenza A (H3N2) in 2017-2018,^28^ with 80,000 US deaths reported by CDC officials,^29^ US coronavirus mortality totals had just reached 80,000 on May 9, 2020.^30^ By then, relative to the 2017-2018 influenza, it was clear that the coronavirus mortality total for the season would be nowhere near 800,000 deaths inferred from the 10-fold mortality overestimation reported to Congress. Even after adjusting for the effect of successful mitigation measures that may have slowed down the rate of coronavirus transmission, it seems unlikely that so many deaths were completely eliminated by a nonpharmaceutical intervention such as social distancing, which was only intended to contain infection transmission, not suppress infections and related fatalities.^31^ Also in early May, 2020, a New York State survey of 1269 COVID-19 patients recently admitted to 113 hospitals found that most of the patients had been following shelter-in-place orders for 6 wk, which raised state officials’ suspicions about social distancing effectiveness.^32^ Still, polls showed the public credited social distancing and other mitigation measures for reducing predicted COVID-19 deaths, and for keeping people safe from the coronavirus.^33,34^

Surprisingly, disproportionate mortality increases in Italian and American health-care facilities during the height of the COVID-19 outbreak were not unique; similar health-care facility crises occurred during the 2016-2017 influenza season in Italy,^35^ and during the 2017-2018 influenza season in the United States.^36^ Yet, these earlier outbreaks did not appear to receive the same intensive media coverage as COVID-19. Although media reports of new coronavirus infections reinforced the public’s belief that the virus was continuing to spread, greater levels of testing may have increased detection of infections that were already prevalent throughout the population. In addition, the accuracy of coronavirus tests rushed into production during the pandemic were unknown.^37^ RT-PCR testing has been in use since the detection of the A (H5N1) influenza virus in 2005,^38^ but a serious limitation of RT-PCR testing is that nucleic acid detection is not capable of determining the difference between infective and noninfective viruses.^39^ Moreover, the CDC modified criteria to record coronavirus mortality by including “probable” and “likely” deaths in the International Classification of Diseases code (ICD) for COVID-19.^40^

By June 21, new daily deaths from the coronavirus dropped to 267 in the United States, a 90% decrease from 2693 daily deaths reported on April 21.^30^ However, confirmed cases in some areas increased as lockdowns lifted,^41^ and total US infections had reached 1,254,055 by June 21.^30^ Several reasons in addition to increased viral transmission could account for case increases. For example, ill people may no longer fear going to hospitals as society reopens,^42^ and coronavirus testing may also result in greater differential diagnosis of SARS-CoV-2 infections from other common respiratory viral infections.^43^ With more reported cases of COVID-19 in younger people following reopening,^44^ CFRs could actually decline due to lower associated mortality risk in this age group. Furthermore, country comparisons of coronavirus CFRs are often confounded by numerous factors,^45^ including health-care differences in case definitions, access to quality treatment and reliable testing, compliance with mitigation measures, and underlying health conditions; demographic differences in age, race, socioeconomic status, and population density; and geo-political differences including climate, seasonality, environmental pollution, social inequities and unrest, personal liberties, public health policies, reliability in reporting valid government statistics of disease, and lifestyle customs that affect physical and mental health, public sanitation, and personal hygiene. Ultimately, with a myriad of uncontrolled confounding factors, a serosurvey of representative samples of a population is a more reliable method to determine the true prevalence of coronavirus infections.

Emerging confounding factors in the United States have also contributed to a rising mortality trend in ILIs such as COVID-19. For example, each year surviving members of the ageing Baby-Boomer cohort of 76 million people born between 1946 and 1964 enter the high-risk category for ILIs, increasing the burden placed on health-care systems.^46^ Also, research shows that a warming trend in the Artic can lead to more extreme winter weather conditions, especially in the Eastern United States,^47^ which may play a role in rising mortality rates from ILIs during the influenza season.

As health authorities responded to the COVID-19 pandemic by implementing lockdowns and other mitigation measures with minimal supporting evidence, scientists warned of “a fiasco in the making,”^48^ Caution was also raised against violations of fundamental principles of science and logic, such as the mistaken assumption that correlation implies causation.^45^ For example, the public’s belief that mitigation measures were responsible for reducing coronavirus mortality may be a post hoc fallacy if lower mortality was actually due to the overestimation of coronavirus deaths. Furthermore, implementing the unconfirmed hypothesis that mitigation measures save lives in vulnerable populations, and rejecting the null hypothesis that assumes no life-saving effect exists, is a type I error in hypothesis testing.^49^ The null hypothesis does not assume a priori knowledge. Therefore, before implementing mitigation measures that incur severe costs, the onus is on mitigation proponents to formally reject the null hypothesis by justifying claims of life-saving benefits. Additionally, education in principles of basic research methods is essential for consumers of public health research, and there is a need to increase instruction in the science and logic of research methods in general education curricula.^50^ More research of nondrug mitigation interventions is also urgently needed to prevent COVID-19, especially in vulnerable populations.^51^

Scientists also warned of public health decisions made without reliable data of infection prevalence within the population.^45,48^ Lacking valid input data due to insufficient testing for disease prevalence, statistical modeling methods often relied on speculative assumptions, producing fearful predictions of increased mortality, which have often proved unreliable.^52^ A systematic review found that most diagnostic and predictive models for COVID-19 lack rigor, have a high risk of selection bias, and are likely to have lower predictive performance in actual practice compared with optimistic reports published in the research literature.^53^

A revised version of a non–peer-reviewed study on COVID-19 antibody seroprevalence in Santa Clara County, California, found that infections were many times more prevalent than confirmed cases.^54^ As more serosurveys are conducted throughout the country, a nationally coordinated COVID-19 serosurvey of a representative sample of the population is urgently needed,^55^ which can determine if the national IFR is low enough to expedite an across-the-board end to restrictive mitigating measures. Plans for a national US serosurvey were announced in April 2020 by the National Institutes of Health, to be conducted by NIAID and the National Institute of Biomedical Imaging and Bioengineering (NIBIB), with the assistance of the National Center for Advancing Translational Sciences (NCATS) and the National Cancer Institute (NCI).^56^ Of relevance, nationwide mitigation measures, such as lockdowns, social distancing, and shelter-in-place orders, were not implemented during the 2017-2018 influenza with 45 million US illnesses reported by the CDC.^57^ Neither were mitigation measures implemented during the 2009 influenza, with reported estimates adjusted for underreported hospitalizations of approximately 60.8 million US cases, ranging between 43.3 million to 89.3 million cases.^58^

### Fear and Collateral Damage

Psychological adverse effects, such as anxiety, anger, and posttraumatic stress, have been linked to restrictive public health mitigation measures due to isolation, frustration, financial loss, and fear of infection.^59,60^ A June 8, 2020, survey from the Association for Canadian Studies found that fear of contracting the coronavirus affected 51% of the Canadian population, compared with 56% of the US population.^61^ Venturing out into public during the reopening phase of the lockdown was stressful to 50% of Canadians compared with 56% of Americans. A second wave of the virus was also expected by 76% of Canadians and 64% of Americans. Furthermore, the possibility exists that yet another novel virus could emerge, potentially reigniting a perpetual process of unfounded fear and unnecessary lockdowns if mortality estimations are not properly vetted.

Fear, in contrast to moral civic duty and political orientation, was shown to be a more powerful predictor of compliance with mitigating behavior in response to a viral pandemic, but with decreasing well-being and poorer decision-making.^62^ Studies have shown that fear impairs performance of cognitive tasks through debilitating anxiety and worry.^63^ Even if a threat ceases to exist, prolonged fearful avoidance of threats is maladaptive and restricts a return to normal social interaction and productivity.^64^ For example, after the outbreak of SARS had ended in 2004, avoidance behavior continued to restrict people’s social interactions and prevented people from returning to work.^65^

Exaggerated levels of fear were driven by sensationalist media coverage during the COVID-19 pandemic.^45,66,67^ And yet, while the public was ordered to lockdown, overall costs and benefits to society from severe mitigation measures had not been assessed.^45^ Fear of infection also prevented people from seeking needed health-care services in hospitals during the pandemic.^68^ The ethics of implementing fear-based public health campaigns needs to be reevaluated for the potential harm these strategies can cause.^69^ Dissemination of vital health information to the public should use emotionally persuasive messaging without exploiting and encouraging overreactions based on fear.

In addition, legal and ethical violations associated with mitigation of pandemic diseases were previously investigated by the Institute of Medicine in 2007.^70^ People should have the right to full disclosure of all information pertinent to adverse impacts of mitigation measures during a pandemic, including information on legal and constitutional human rights issues,^45^ and the public should be guaranteed a voice in a transparent process as authorities establish public health policy.

Last, severe mitigating measures during the COVID-19 pandemic caused considerable global social and economic disruption.^71^ Enforced lockdowns increased domestic violence, closed businesses and schools, laid off workers, restricted travel, affected capital markets, threatened the security of low-income families, and saddled governments with massive debt. Between February and April 2020, US unemployment rose from 3.5%, the lowest in 50 years, to 14.7%.^72^ A recession in the United States was also officially declared in June 2020 by the National Bureau of Economic Research, ending 128 months of historic economic expansion. Of relevance, economic downturns are associated with higher suicide rates compared with times of prosperity, and increased suicide risk may be associated with economic stress as a consequence of severe mitigation measures during a pandemic.^73^ Relapses and newly diagnosed cases of alcohol use disorder were also predicted to increase due to social isolation, and harmful drinking in China increased 2-fold following the COVID-19 outbreak.^74^ As a global natural experiment, psychological outcomes from restrictive interventions in the COVID-19 pandemic require further investigations.^75^

**FIGURE 2:**
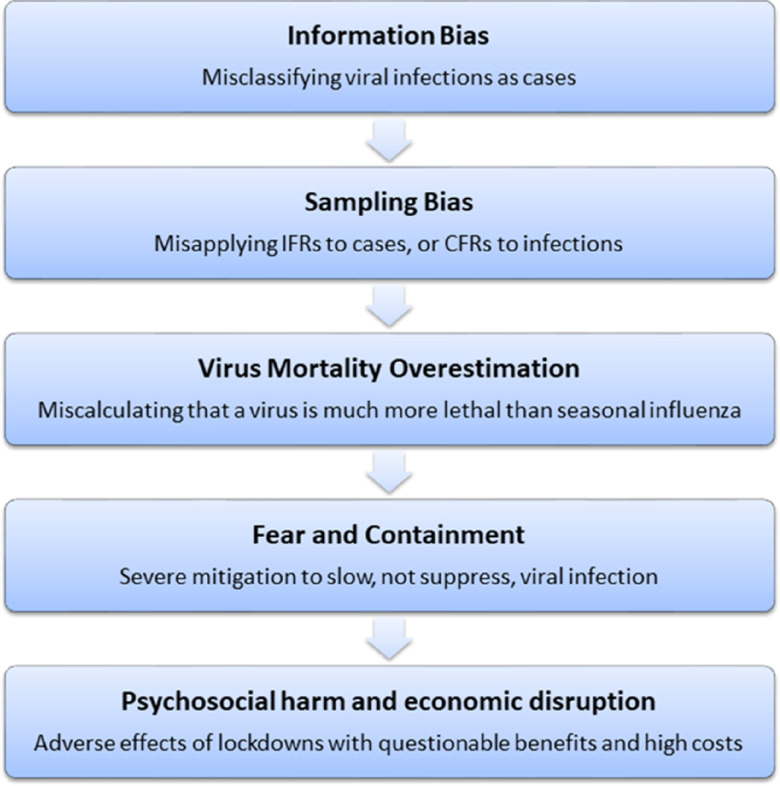
Biases and Potential Related Effects of Virus Mortality Overestimation.

Public health lessons learned during the COVID-19 pandemic contribute knowledge and insights that can be applied to prevent future public health crises.^76^ Figure 2 shows a flow chart that summarizes biases and potential effects of viral mortality overestimation observed in a pandemic. Failure to intervene at the source of the problem, at the upstream levels of information bias and sampling bias, can allow fear to rapidly escalate and may cause an overactive response that produces severely harmful collateral damage to society.

## CONCLUSIONS

Sampling bias in coronavirus mortality calculations led to a 10-fold increased mortality overestimation in March 11, 2020, US Congressional testimony. This bias most likely followed from information bias due to misclassifying a seasonal influenza IFR as a CFR, evident in a NEJM.org editorial. Evidence from the WHO confirmed that the approximate CFR of the coronavirus is generally no higher than that of seasonal influenza. By early May 2020, mortality levels from COVID-19 were considerably below predicted overestimations, a result that the public attributed to successful mitigating measures to contain the spread of the novel coronavirus.

This article presented important public health lessons learned from the COVID-19 pandemic. Reliable safeguards are needed in epidemiological research to prevent seemingly minor miscalculations from developing into disasters. Sufficient organizational quality assurance procedures should be implemented in public health institutions to check, catch, and correct research biases and mistakes that underestimate or overestimate associated risks of disease and mortality. Particularly, the denominator of fatality rates should clearly define the group to whom fatalities apply. Public health campaigns based on fear can have harmful effects, and the ethics of such campaigns should be reevaluated. People need to have a greater voice in a transparent process that influences public health policy during an outbreak, and educational curricula should include basic research methods to teach people how to be better consumers of public health information. The public should also be fully informed of the adverse impacts on psychological well-being, human rights issues, social disruption, and economic costs associated with restrictive public health interventions during a pandemic.

In closing, nations across the globe may fearfully anticipate future waves of the coronavirus pandemic, and look bleakly toward outbreaks of other novel viral infections with a return to severe mitigation measures. However, well-worn advice from a famous aphorism by the poet philosopher George Santayana should be borne in mind, which is relevant to public health lessons learned in this article: “Those who cannot remember the past are condemned to repeat it.”^77^
